# Polarization-Tunable Photoelectrochemistry at Individual Anisotropic ReS_2_ and Its Homostructure Interfaces

**DOI:** 10.1021/acs.jpclett.6c00693

**Published:** 2026-04-14

**Authors:** Pelumi Adanigbo, Aidan C. Malloy, Maha Laiq, Lucas Wang, Alec G. Freschlin, Patrick M. Vora, Yun Yu

**Affiliations:** † Department of Chemistry and Biochemistry, 3298 George Mason University, Fairfax, Virginia 22030, United States; ‡ Department of Physics and Astronomy, George Mason University, Fairfax, Virginia 22030, United States; § Quantum Science and Engineering Center, George Mason University, Fairfax, Virginia 22030, United States

## Abstract

Light polarization offers a powerful yet underexplored handle to control photoelectrochemical processes in two-dimensional (2D) semiconductors. Here, we demonstrate polarization-tunable photoelectrochemistry at anisotropic 2D ReS_2_ interfaces, providing a new strategy to manipulate light-driven charge dynamics. Using scanning electrochemical cell microscopy (SECCM) under controlled photoexcitation, we systematically probe how incident wavelength, layer thickness, and van der Waals stacking govern polarization sensitivity at the nanoscale. The dichroic ratio increases by ∼50% as the excitation wavelength approaches the band edge and decreases systematically with thickness, while the polarization phase shift grows with both wavelength and layer number due to ReS_2_ birefringence. By stacking ReS_2_ layers with controlled twist angles and thickness contrasts, we achieve programmable junction behavior ranging from nearly isotropic responses in 90°-twisted bilayers to layer-dominant anisotropy dictated by photogeneration balance. For arbitrary twist angles, the phase shifts of the individual layers add coherently, enabling predictive control of the angular response. This work establishes light polarization as a precise and versatile control knob for nanoscale photoelectrochemistry, offering a new paradigm for designing optoelectronic and photocatalytic devices with intrinsic polarization selectivity.

## Graphical abstract

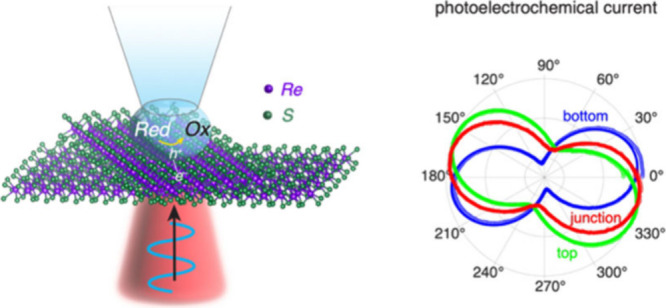

Photocatalysis offers a promising approach to address global challenges in energy, sustainability, and environmental remediation.^1,2^ Research has predominantly concentrated on enhancing metrics, such as quantum efficiency,^3,4^ stability,^5^ and catalytic reactivity,^4–6^ through diverse methodologies. Despite performance improvements, methods that enable real-time control of the reaction rate during the operation remain limited. In this context, incorporating light polarization, a fundamental illumination parameter, presents a compelling opportunity to dynamically modulate photoelectrochemical processes. While this approach has made notable progress in piezoelectric^7^ or ferroelectric materials,^8,9^ the primary focus is often on enhancing charge separation and migration, which frequently requires the application of additional fields.^10–13^

Two-dimensional (2D) transition metal dichalcogenides (TMDs) exhibit promising potential as photocatalysts due to their favorable bandgap, extensive surface area, and minimized migration distance for photogenerated carriers to reach the electrolyte.^14,15^ Low-symmetry 2D TMDs are characterized by their distorted crystal structures and in-plane anisotropy. This structural anisotropy has enabled polarization-modulated light–matter interactions, including optical absorption,^16,17^ vibrational modes,^18,19^ exciton dynamics,^14,20,21^ and carrier mobility.^22^ Among these anisotropic TMDs, ReS_2_ exhibits a distinctive distorted 1T crystal structure.^22^ The distortion leads to the formation of Re–Re clustered chains aligned along the crystallographic *b* axis (Figure 1a). This low-symmetry triclinic structure breaks the in-plane isotropy typically seen in other TMDs and gives rise to strong anisotropy in its optoelectronic properties.^16–21^ These properties persist even in multilayer or bulk forms due to weak interlayer coupling,^23^ making ReS_2_ an exceptional model system to explore the potential of polarization control in interfacial light-driven chemical conversions. Despite its intensive use in diverse photo-sensing applications,^17,24,25^ harnessing polarization control over ReS_2_ in the realm of photocatalysis^26,27^ remains largely untapped. Parasuraman et al. showed that aligning light polarization with the Re chain from band-edge excitons enhances the axial photodegradation of organic dyes on ReS_2_.^14^ Nevertheless, the roles of fundamental factors, such as light wavelength and material thickness, in influencing the reaction sensitivity to polarization remain unclear.

**Figure 1:**
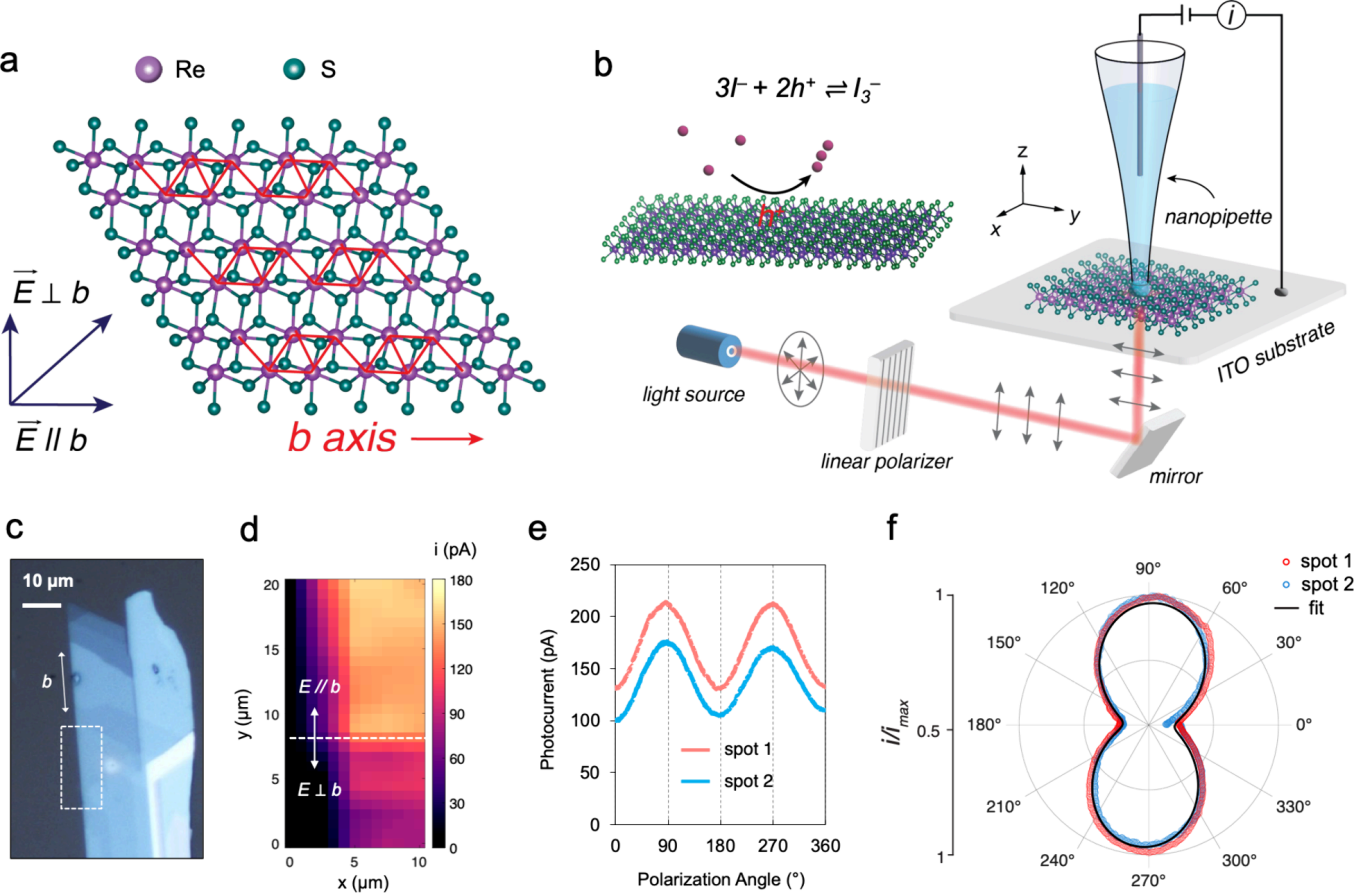
(a) Schematic top view of the anisotropic layered structure of the ReS_2_ crystal. (b) Schematic of photo-SECCM integrated with an inverted microscope, where excitation polarization is controlled by rotating the linear polarizer. (c) Optical image of a 13 nm thick ReS_2_ flake on an ITO substrate. (d) 20 × 10 μm photoelectrochemical current map of the highlighted region in panel c, obtained at 0.4 V vs Ag/AgI with 290 mW/cm^2^ 650 nm illumination. The light polarization was rotated by 90° when the SECCM probe reached *y* = 8 μm (horizontal dash line). (e) Current vs polarization angle traces obtained at two different spots of the ReS_2_ flake under 290 mW/cm^2^ 650 nm excitation and 0.4 V vs Ag/AgI bias. (f) Polar plots of the normalized photocurrent of the two curves in panel e and the fit to eq 1.

van der Waals (vdW) heterostructures formed by deterministic stacking of 2D layers^28,29^ offer unprecedented control over interfacial chemistry, where parameters such as stacking sequence^30,31^ and twist angle^32,33^ have been precisely tuned to engineer new emergent properties. While vdW systems based on isotropic materials^34,35^ have been widely explored, the integration of anisotropic materials into these stacks remains largely untapped, particularly in the context of applying polarization control for interfacial redox chemistry. In contrast to the modulation of band alignment^36,37^ and interlayer hybridization,^38^ stacking anisotropic 2D layers offers a distinctive approach to modifying the intrinsic in-plane anisotropy. Although this strategy has unveiled emergent phenomena, including Raman modes,^39^ optical reflectance,^40,41^ chirality,^42^ and photosensitivity,^43,44^ its potential role in regulating photodriven chemical transformations remains unexplored.

In this study, we have employed photomodulated scanning electrochemical cell microscopy (photo-SECCM)^34,45,46^ for investigations of individual ReS_2_ layers and its vdW homostructures. Such spatially resolved measurements reveal critical local photoactivities that are inaccessible in ensemble measurements.^15,47–51^ In a typical SECCM configuration,^34,35,45–50^ a nanodroplet of electrolyte formed at the tip of a nanopipette contacts a sample surface, establishing a confined electrochemical cell at each pixel (Figure 1b). By integrating linearly polarized light into the SECCM setup, we obtained quantitative, site-specific correlation between the polarization angle, crystal orientation, and local photoelectrochemical activity.

Exfoliated ReS_2_ flakes, with thicknesses ranging from a few layers to several tens of nanometers, were placed onto transparent indium tin oxide (ITO) substrates. The crystallographic *b*-axis orientation was determined based on the longer straight edge^14^ of the cleaved ReS_2_ crystal (Figure 1c) and subsequently confirmed using polarization-resolved Raman spectroscopy (Figure S1). A linearly polarized light beam is introduced into the bottom of the ReS_2_ flakes through an inverted microscope, ensuring complete illumination of the entire flake. The light spot size (∼200 μm) significantly exceeds the size of the ReS_2_ flakes and the SECCM probe. Using nanopipettes filled with electrolytes containing 0.1 M KI and 0.01 M I_2_, we observed a consistent photocurrent associated with the photooxidation of iodide species, 3I^–^ – 2e^–^ → I_3_^–^, as depicted in Figure S2. The sub-micrometer nanopipettes employed in this study are significantly smaller than the dimensions of the ReS_2_ flakes and the light spot. SECCM mapping (Figure 1d) was conducted on the highlighted region in Figure 1c to visualize the photooxidation rate as the polarization is modulated. No measurable photocurrent was observed at the ITO surface. In contrast, the ReS_2_ surface exhibited a clear photoactivity. While the polarization is perpendicular to the *b* axis of the ReS_2_ flake (*E*⊥*b*, below the dashed line), a reasonably uniform photocurrent of approximately 90 pA was obtained across the flake. Upon polarization rotation by 90° such that it becomes aligned with the *b* axis (*E*∥*b*, above the dashed line), the photocurrent experienced a 2-fold increase. The pronounced contrast observed in SECCM mapping suggests a strong correlation between photoelectrochemical activity and light polarization. Notably, this polarization effect is primarily attributed by the lower energy band-edge exciton, as highlighted in the previous report.^14^

A more systematic examination of the angular-dependent photoactivity was conducted by monitoring the photocurrent of iodide oxidation as the polarizer was rotated from 0° to 360° with respect to the *b* axis of the flake at a rate of 5°/s. The raw data (Figure 1e) obtained by the nanopipet positioned at two arbitrary spots of the ReS_2_ flake demonstrate periodic modulation of the photocurrent with the polarization angle. To facilitate visualization of the dependence, the current traces were normalized by the maximum current and presented in a polar plot (Figure 1f). A dumbbell-shaped curve exhibiting an elongated axis consistent with the *b* axis of the ReS_2_ flake was observed. The highest current was recorded when the polarized light (near 90° and 270°) was parallel to the *b* axis of the flake, while the lowest responses were observed when the polarization was perpendicularly aligned with the *b* axis of the flake (near 0° and 180°). This is consistent with the maximum photoabsorption occurring with polarization parallel to the Re–Re chains (*b* axis) and is minimized when perpendicular. To further quantify the angular dependence, the experimental data were fitted with a simplified model (eq 1).

1 $$i\left(\theta \right)=A\cos^{2}\left(\theta -\varphi \right)+B$$

*A* and *B* represent the anisotropic factor (polarization-dependent) and the isotropic factor (polarization-independent), respectively. θ denotes the polarized angle, and φ represents the angle offset relative to the crystallographic reference axis of the flake. Through the fitting process, we obtain the dichroic ratio, *R* = (*A* + *B*)/*B*, to quantify the strength of the photoelectrochemical anisotropy. This parameter is equivalent to the ratio of the maximum to minimum photocurrent, as θ varies. The angle offset, φ, is determined by the alignment of the ReS_2_ flake relative to the 0° polarization orientation. As will be elaborated in subsequent sections, φ is also influenced by the phase shift resulting from light transmission through a birefringent crystal.^52^ The two traces can be fitted to eq 1 by using the same parameters, demonstrating a consistent angular dependence at various locations within the same ReS_2_ flake.

Our control measurements on an isotropic MoS_2_ flake using the same experimental conditions showed no polarization dependence of the photocurrent (Figure S3), supporting that the polarization-tunable photocurrent observed in Figure 1e originates from the intrinsic in-plane anisotropy of ReS_2_ rather than an experimental artifact.

To elucidate the role of carrier transport in the polarization-dependent photoelectrochemical activity, we measured the direction-dependent transfer characteristics of a 37 nm ReS_2_ flake placed onto an insulating substrate. Figure 2a illustrates that the graphite flakes electrically contact the ReS_2_ flake. Probe 1–2 was positioned along the *b* axis of ReS_2_, while probe 3–4 was positioned perpendicular to the *b* axis. Figure S4 illustrates the direction-dependent photocurrents at a fixed voltage between the two contacts, while the ReS_2_ flake is illuminated with a 650 nm bottom light. The sheet photoconductance was evaluated using eq 2

2 $$G=\frac{I_{p}}{V}\frac{L}{W}$$

where the length between two electrodes is *L* and the width of the current path is *W*. *I*_p_ and *V* are the photocurrent and applied voltage, respectively. Figure 2b shows polarization-dependent *G* along the two directions, suggesting that higher photoconductivity is obtained along the *b* axis than crossing the *b* axis. This result aligns with the previous report,^22^ indicating that carrier mobility is higher along the *b* axis, where the Re–Re chains facilitate enhanced in-plane conduction. When the photoconductance at each polarization angle was normalized to its maximum value (Figure 2c), however, both orientations showed almost identical angular dependence, exhibiting the same shape and similar dichroic ratio. This behavior suggests that the polarization response originates primarily from anisotropic optical absorption and carrier generation rather than directional differences in carrier transport. The role of in-plane transport anisotropy appears negligible, particularly considering that the current measured by SECCM is predominantly governed by out-of-plane charge transfer. The polarization sensitivity in photoelectrochemistry is therefore tuned through the anisotropy of optical absorption and carrier generation rather than charge mobility modulation.

**Figure 2:**
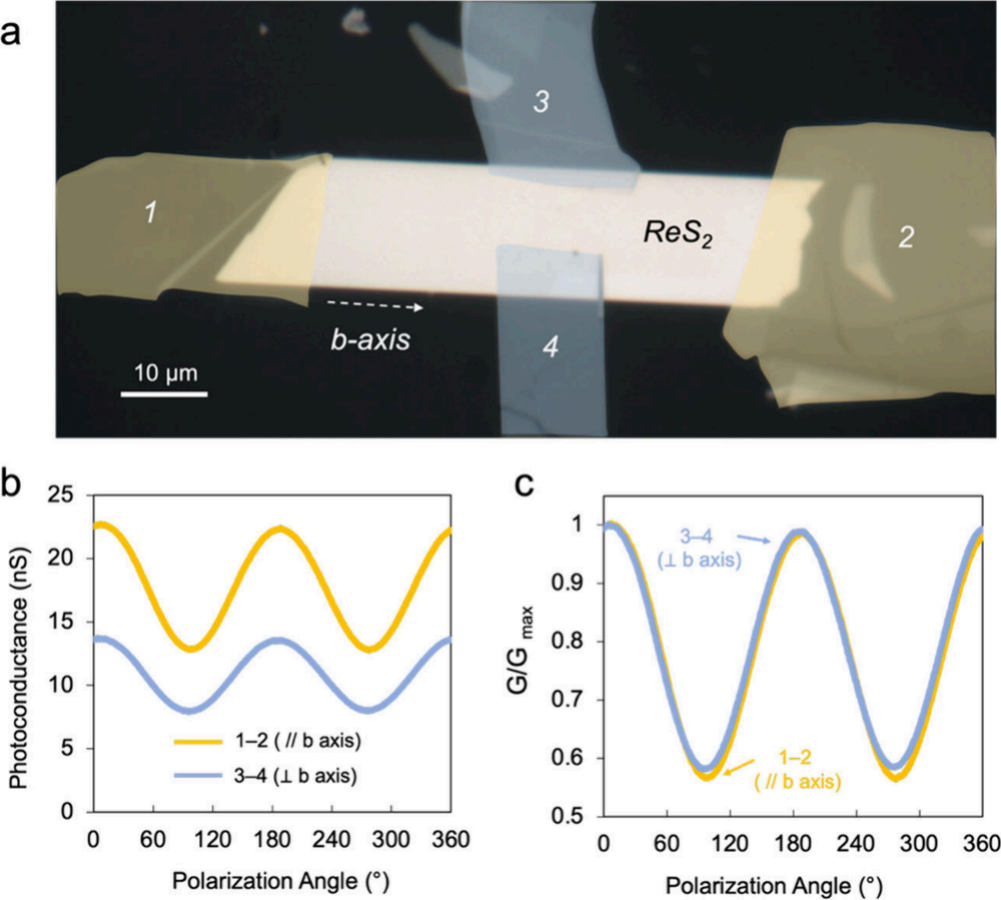
(a) Optical image of a 37 nm ReS_2_ flake connected to four graphite contacts (color highlighted) with probes 1–2 and 3–4 positioned along and perpendicular to the *b* axis of ReS_2_, respectively. (b) Sheet photoconductances obtained from the four probes under linearly polarized light as a function of the polarization angle. (c) Normalized photoconductance plot of panel b.

The effect of the electrical potential on the polarization-dependent behavior was investigated by using local voltammograms (Figure S5a) and amperometric data spanning a potential range (Figure S5b). The normalized current vs angle curves are nearly indistinguishable, indicating that the ability to control the photocurrent using polarization is largely unaffected by the applied potential.

With regard to the effects of excitation power, Figure S6 revealed that higher light intensity results in enhanced photon flux and, thus, a higher magnitude of photocurrent. However, the polarization dependence and associated dichroic ratio remain constant within the linear regime of photocurrent versus intensity dependence. The anisotropy is preserved and unaffected by low to moderate intensities, though we anticipate that nonlinear and saturation response at high intensities could obscure or diminish the polarization sensitivity.^53^

To understand the role of the excitation wavelength in photoelectrochemical anisotropy, we probed the angular-dependent photoelectrochemical activity at ReS_2_ surfaces with three different wavelengths (550, 600, and 650 nm). Although the bandgap of ReS_2_ is >900 nm, we observed minimal photocurrent with excitation wavelengths of >700 nm due to limited photoabsorption. Therefore, 650 nm was selected as the highest wavelength to minimize errors in analyzing weak signals. After normalizing the photocurrents for I^–^ oxidation (Figure S7) by the maximum current, we present the polar plots of the ReS_2_ responses for different thicknesses under various excitation wavelengths in Figure 3a. The effect of wavelength is manifested in affecting both the dichroic ratio, *R*, and the angular offset, φ. As the wavelength increases across all thicknesses, the polar plot elongates and deviates more strongly from a circle, indicating stronger polarization anisotropy and a higher dichroic ratio. Meanwhile, the polar plot of higher wavelengths exhibits a rotated dumbbell shape compared to that of the sample orientation, suggesting a larger phase shift in the polarization dependence.

**Figure 3:**
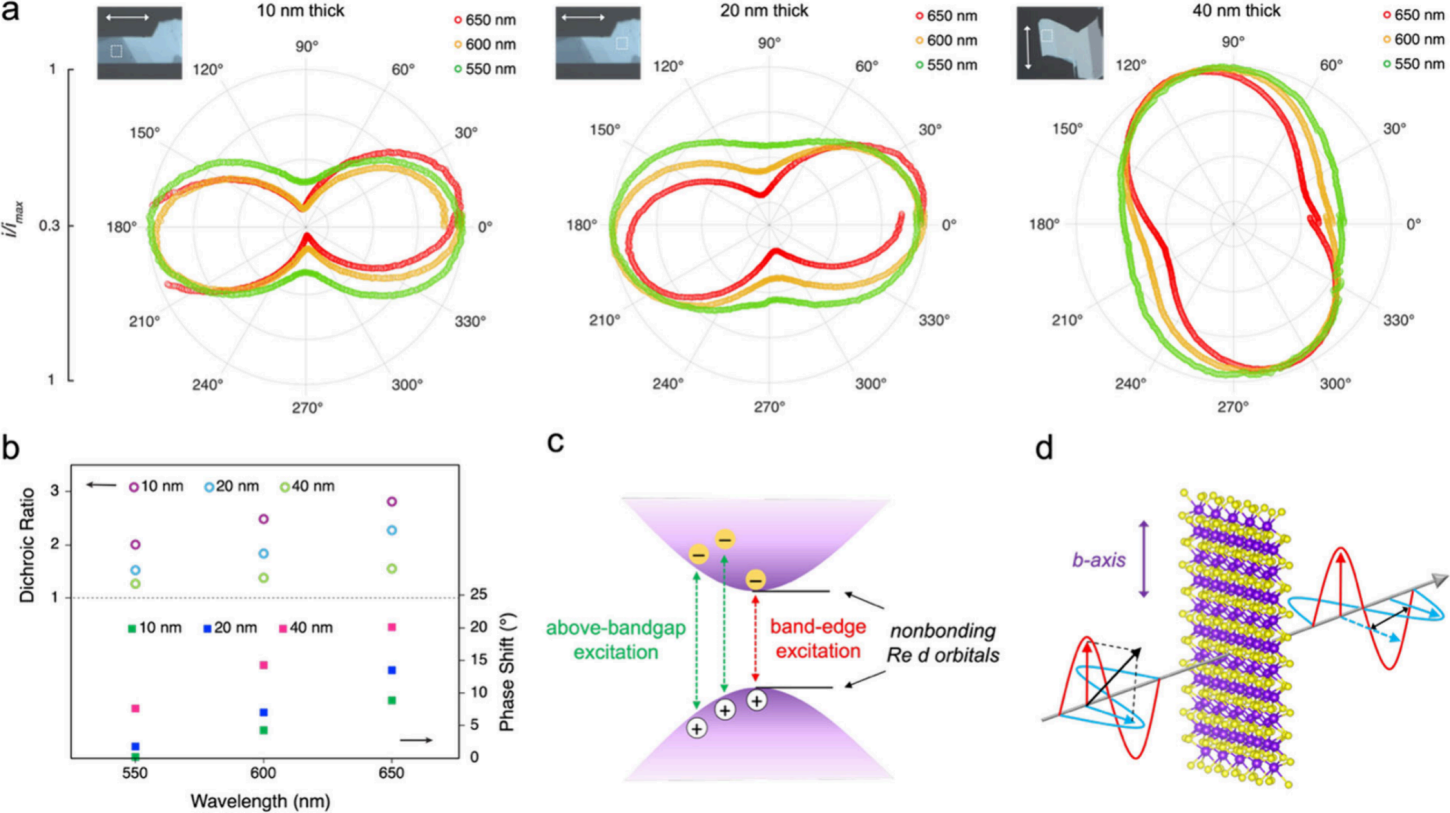
(a) Polar plots of normalized photoelectrochemical current response of ReS_2_ flakes with various thicknesses under different photoexcitation wavelengths. Each inset shows the optical image of the region of interest and direction of the *b* axis. (b) Dichroic ratio and phase shift of different thickness ranges as a function of the wavelength. (c) Energy band diagram of ReS_2_ illustrating two types of electronic transition. (d) Schematic diagram illustrating the phase shift as linearly polarized light transmits through a ReS_2_ flake.

The upper panel of Figure 3b illustrates that the dichroic ratio can be significantly enhanced by 50% when the wavelength is increased from 550 to 650 nm. This trend of enhanced polarization sensitivity with increasing wavelength is observed throughout the entire range of thickness investigated in this study. This enhancement can be attributed to the nature of the band-edge transitions and their anisotropic orbital contributions in ReS_2_. The electronic states near the band edge are dominated by non-bonding Re 5d orbitals,^54,55^ whose directional overlap along the Re–Re chains gives rise to strong linear dichroism in both optical absorption and photocurrent response. At longer wavelengths near the band-edge region (e.g., 650 nm), the transitions primarily involve the Re 5d states, which leads to an amplified dichroic response. Conversely, at shorter wavelengths (e.g., 550 nm), above bandgap excitations involve transitions from deeper valence band states with a mixed S 3p and Re 5d character. These transitions are relatively delocalized and less sensitive to in-plane anisotropy, resulting in a smaller dichroic ratio. This effect is illustrated in Figure 3c, suggesting that band-edge excitation preferentially promotes carriers associated with the anisotropic Re–Re bonds. Similar trends have been reported in polarization-resolved absorption measurements of few-layer ReS_2_.^17^

In addition to the enhancement of the dichroic ratio, a pronounced increase in the phase shift in the polarization-dependent responses is also observed as the excitation wavelength approaches the band-edge region (lower panel of Figure 3b). As illustrated in Figure 3d, when polarized light passes through ReS_2_, its components along and perpendicular to the *b* axis encounter varying refractive indices, leading to the accumulation of a phase delay. The phase shift, ΔΦ, was determined from the angular offset, φ, extracted from the data fit using eq 1, and the geometric alignment of the ReS_2_ flake was measured from the optical images. The determined phase shift reflects the intrinsic birefringence of ReS_2_ arising from the difference in refractive indices along the *b* axis (*n*_1_) and the perpendicular direction (*n*_2_). The relationship between the measured phase shift and the optical anisotropy can be expressed as

3 $$\Delta \Phi =\frac{2\pi }{\lambda }\left(n_{1}-n_{2}\right)d$$

where λ is the wavelength and *d* is the sample thickness. As the excitation wavelength increases from 550 to 650 nm, ΔΦ systematically grows due to an increase of *n*_1_ – *n*_2_. Although the 1/λ factor tends to reduce ΔΦ at longer wavelengths, the experimental observation of an increasing phase shift demonstrates that *n*_1_ – *n*_2_ rises strongly near the band-edge excitation. This trend corresponds to a greater difference in the propagation velocity of light through the two crystallographic orientations, reflecting the directional coupling of photons to the anisotropic electronic orbitals. Such wavelength-dependent birefringence is a hallmark of low-symmetry layered materials^56,57^ and provides an additional signature in their polarization-tunable photoelectrochemical behavior.

To this end, we can interpret the concurrent increase in the dichroic ratio and phase retardance with the wavelength in the context of the Kramers–Kronig relations,^58^ which link the real and imaginary components of the complex refractive index. In anisotropic ReS_2_, the polarization-dependent absorption coefficient (imaginary part, *k*) and refractive index (real part, *n*) are intrinsically coupled through these relations. According to the Kramers–Kronig framework, an enhancement of absorption anisotropy (Δ*k*) necessarily gives rise to a corresponding dispersion in birefringence (Δ*n*). Therefore, the observed wavelength-dependent dichroism and birefringence are fundamentally correlated.

Our polarization-resolved measurements demonstrate a clear correlation between the flake thickness and the dichroic ratio as well. Within the examined range of 10–40 nm (Figure S8), *R* systematically decreases with increasing thickness at all wavelengths (upper panel of Figure 3b). This indicates a gradual diminution of the polarization sensitivity as the material becomes thicker. We propose that this phenomenon arises from the averaging out of anisotropic absorption due to multiple absorptive and scattering events, resulting in a reduced contrast between orientations parallel and perpendicular to the *b* axis. These findings are supported by our photoabsorption measurement conducted using an inverted microscope (Figure S9), wherein the dichroic ratio of the absorbance also exhibits a monotonically decreasing trend with increasing thickness.

In contrast to the decreasing dichroic ratio, the phase shift (ΔΦ) between the two polarization components increases with the ReS_2_ thickness (lower panel of Figure 3b). This behavior is consistent with the birefringent phase retardation described in eq 3. As the thickness increases, this phase delay becomes larger, resulting in a greater overall phase shift. The trend is consistent with ReS_2_ behaving as a birefringent medium in multilayer form, where the cumulative propagation difference through layers enhances the optical retardation.^59^ Within the thickness range of 10–40 nm, the effective linear birefringence (Δ*n*_eff_) was estimated using the ΔΦ vs *d* relationship in eq 3. As the excitation wavelength increases from 550 to 650 nm, Δ*n*_eff_ exhibits a systematic increase from 0.38 to 0.66. We emphasize that these values represent an effective birefringence manifested in photoelectrochemistry rather than a purely intrinsic material property of the ReS_2_ lattice.

For extremely thin ReS_2_ flakes (<10 nm), the photocurrent signal was insufficiently strong to be reliably measured using the SECCM setup, likely attributed to limited light absorption and a diminished active volume for carrier generation. For thicknesses exceeding 40 nm, we anticipate that optical interference may complicate the thickness dependence, as the optical path length becomes comparable to a substantial portion of the excitation wavelength. Future work will systematically investigate this regime by integrating our SECCM measurement with optical modeling of interference-induced modulation.

Having established how intrinsic factors govern the dichroic ratio and phase shift in ReS_2_, we next extended our study to the active control of polarization-dependent photoelectrochemical behavior through structural engineering. Specifically, we employ mechanical vdW assembly^29,32^ to construct ReS_2_ homostructures with controlled twist angles, enabling systematic modulation of the in-plane anisotropy at the junction. We demonstrate that the anisotropy in the photoelectrochemical response can be tuned by the layer thickness and twist configurations.

First, we prepared ReS_2_ homostructures by stacking two ReS_2_ layers with their *b* axes rotated by 90° relative to each other, resting on a graphene bottom contact for efficient photocurrent collection (Figure 4a). This configuration results in the vector superposition of two orthogonally aligned anisotropic axes, causing their angular-dependent photocarrier generation to be out of phase. Analogous to destructive interference, this arrangement effectively suppresses the overall optical anisotropy of the stacked structure. By positioning the SECCM probe at each region of the ReS_2_ homostructure surface, we independently measured the angle-dependent photocurrent associated with iodide oxidation under 650 nm excitation. In Figure 4c, the polar plots of the photocurrent responses from the top (green) and bottom (blue) layers reveal fast axes that are oriented perpendicular to each other, as expected. Notably, at the junction region (pink), the anisotropic response is significantly diminished, yielding an almost isotropic behavior characterized by a semicircle.

**Figure 4:**
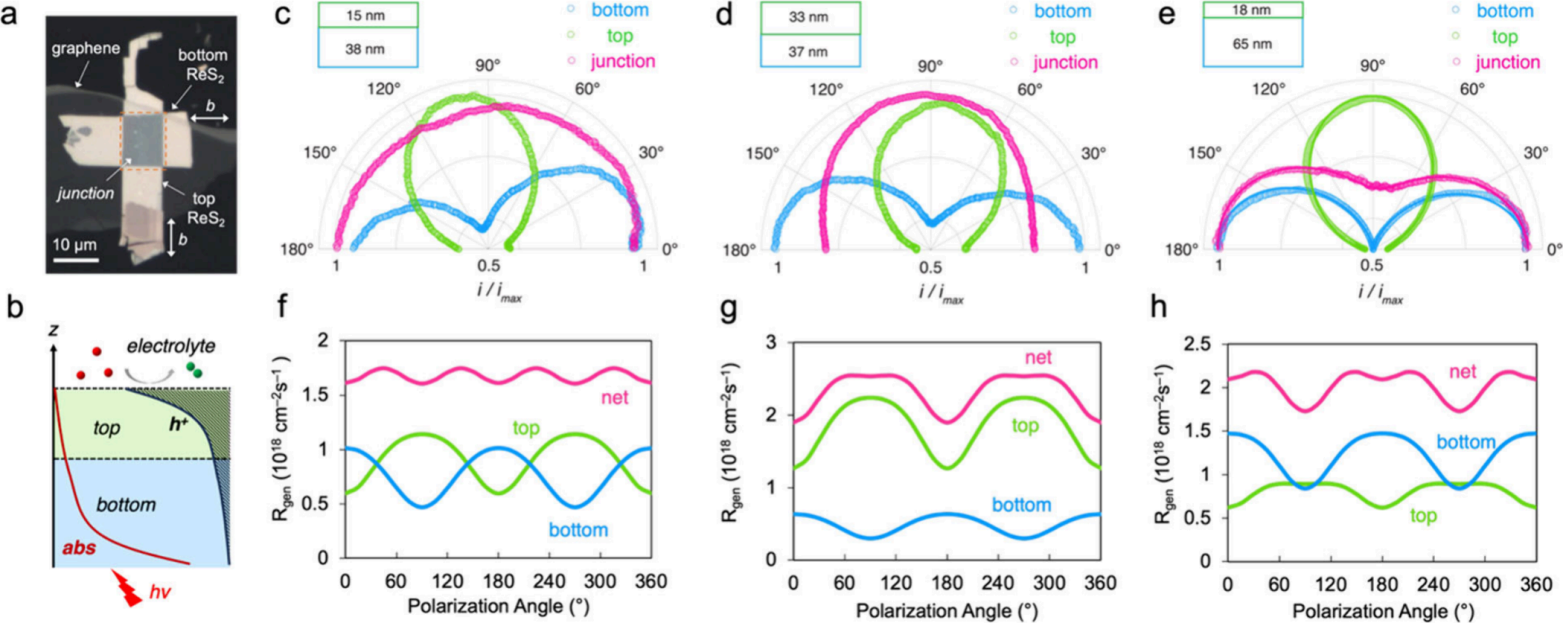
(a) Optical image of a vertically aligned ReS_2_ homostructure on a graphene bottom contact. (b) Schematics illustrating photoabsorption attenuation and hole accumulation within the space charge layer. (c–e) Polar plots depicting the normalized photoelectrochemical current of vertically aligned ReS_2_ homostructures with 650 nm excitation: (c) 15 nm (top) and 38 nm (bottom), (d) 33 nm (top) and 37 nm (bottom), and (e) 18 nm (top) and 65 nm (bottom). (f–h) Simulated photocarrier generation rates (*R*_gen_) at the ReS_2_ homojunctions in panels c–e, along with the respective contribution of the top and bottom layers under a photon flux of 10^19^ cm^–2^ s^–1^.

Unlike other twisting-induced optical phenomena, such as moiré excitons,^60,61^ the strong interlayer coupling between ReS_2_ layers is not required for tuning the photoelectrochemical anisotropy. Figure 4b illustrates that the angle-dependent carrier generation that is out of phase in the two layers collectively dictates the behavior of the junction. Due to our bottom illumination configuration, the bottom layer absorbs more photons and generates more carriers. However, the interfacial photoreaction occurs at the top surface, mediated by the holes accumulated near the electrolyte–semiconductor interface under an anodic bias. Consequently, only the carrier generation within the space-charge layer^62^ should be considered for the calculation of the photocurrent. The space-charge layer is predominantly located in the top ReS_2_ and extends partially to the bottom ReS_2_. We simulated the carrier generation rate at the junction region with simulation details provided in section 3 of the Supporting Information. As depicted in Figure 4f, the two antiphase components with comparable amplitudes lead to a net effect that becomes largely insensitive to the polarization angle.

As demonstrated in Figure 4c, achieving net cancellation requires a thicker bottom layer because of its smaller share of the space-charge region. When the two layers have comparable thickness (Figure 4d), the photoabsorption at the junction becomes nearly isotropic (Figure S10). Nevertheless, the photoelectrochemical current exhibits some degree of anisotropy and is governed by the top layer. As revealed in Figure 4g, our calculation shows a higher weight in the top layer due to more holes generated there participating in the photoreaction. Conversely, the situation can be reversed by a significantly thicker bottom layer, producing a net result at the junction that resembles the bottom layer’s pattern (Figure 4e). Due to the dominating light absorption and carrier generation (Figure 4h), the polarization dependence of the junction is determined by the bottom layer. When the top layer is thicker, it further enforces complete alignment of the junction response with its own anisotropy, as confirmed in Figure S11. These thickness-dependent results provide strong support for our hypothesis that polarization sensitivity at the junction is regulated by the balance of photogeneration between the two layers. While the top layer exerts dominant control, tuning the layer thickness offers an additional lever to control the photoelectrochemical anisotropy in vdW heterostructures.

We next discuss the situation of two ReS_2_ layers twisted at an arbitrary angle. Figure 5a shows an optical image of a sample with the *b* axes of the top and bottom layers aligned at 44°. While the response of the homojunction is intuitively expected to be a vector sum of the two angled layers, phase retardation from polarized light traversing the birefringent layers complicates this behavior. Figure 5b presents polar plots of the photocurrent collected from various regions (labeled in Figure 5a) under different wavelength excitations. For the sake of clarity, the *b*-axis direction of each layer is indicated. The angular dependence data exhibit phase shifts, as evidenced by the misalignment between the dumbbell-shaped curves and the *b* axis of each layer. Consistent with previous discussions, longer wavelengths (e.g., 650 nm) induce larger phase retardation.

**Figure 5:**
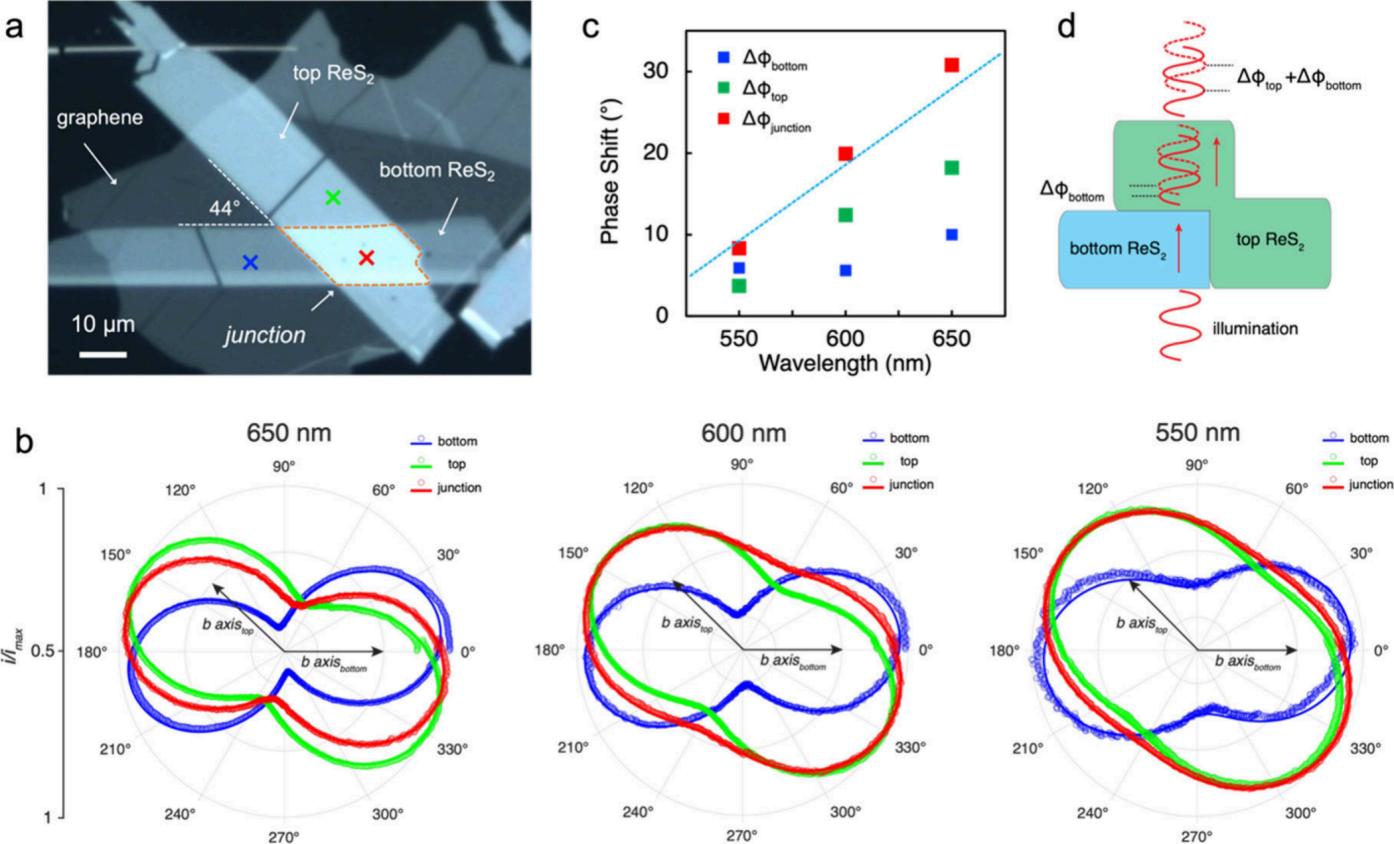
(a) Optical image of a ReS_2_ homostructure composed of a 23 nm (top) and 19 nm (bottom) layer twisted by 44°. (b) Polar plots of the normalized photocurrent under different excitation wavelengths. The solid lines represent the fit of experimental data to eq 1. (c) Phase shift probed at different stacking regions as a function of the excitation wavelength. (d) Schematic diagram illustrating that polarized light transmits through a ReS_2_ homostructure.

The experimental data were fitted to eq 1 to extract the angle offset, φ. Using the optical image as a reference, the phase shifts (ΔΦ) for both the top and bottom layers were calculated after accounting for their respective flake orientations. As per our previous discussion, the top layer primarily affects the polarization dependence of the junction. Based on this assumption, we extracted ΔΦ at the junction, as shown in Figure 5c. Interestingly, the phase shift at the junction (red points) is approximately equivalent to the summation of the phase shifts of the bottom layer and top layer (dashed line) across all wavelengths. This finding suggests a coherent superposition of the polarization-dependent carrier dynamics of both layers, as illustrated in Figure 5d. As light is absorbed and propagated through the artificial heterostructure, the phase distortion at the interface is negligible. This behavior suggests that the interlayer coupling in this system is sufficiently weak to preserve the intrinsic in-plane anisotropy of each ReS_2_ layer. Unlike moiré excitons in TMD bilayers,^39,63^ where twisting primarily modulates the potential of valley polarized excitons in a strongly coupled system, our study on large-angle twisted ReS_2_ stacks exploits the intrinsic in-plane anisotropy to control the photoelectrochemical activity. Our findings align with recent studies showing that twist-induced optical anisotropy in ReS_2_ is unaffected by interlayer coupling.^40^

In conclusion, we have demonstrated the first photo-SECCM study of polarization-dependent photoelectrochemistry in anisotropic 2D ReS_2_ photoelectrodes, providing unprecedented spatial and angular resolution of the local photoresponse. Wavelength-dependent studies demonstrate that band-edge illumination enhances dichroism and induces larger birefringence-driven phase shifts, while increasing flake thickness suppresses dichroic contrast but amplifies phase retardation, offering practical handles to tune both intensity and phase response in nanoscale devices. vdW-assembled homostructures establish a new route to engineer polarization-dependent photoelectrochemistry through twist-angle and thickness modulation. The coherent addition of phase shifts from constituent layers enables predictable angular control, highlighting a versatile design strategy for layered 2D heterostructures. Taken together, these results elucidate the microscopic mechanisms of polarization-driven photoelectrochemistry, provide a generalizable framework for designing anisotropic vdW photoelectrodes, and open new opportunities for polarization-resolved photodetectors, optoelectronic devices, and tailored photochemical energy conversion systems.

## Supplementary Material



## Notes

Information related to experimental methods, Raman characterization, raw SECCM and photoconductance data, AFM images of ReS_2_ flakes, optical absorbance data, and calculation of the generation rate at a ReS_2_ junction (PDF)
