# Supplementary material for: Polarization-Tunable Photoelectrochemistry at Individual Anisotropic ReS2 and Its Homostructure Interfaces
Source: J Phys Chem Lett. 2026 Apr 14;17(16):4861–9. doi: 10.1021/acs.jpclett.6c00693 (PMC13112444; doi:10.1021/acs.jpclett.6c00693)
Supplement: Supplementary file 1 [file jz6c00693_si_001.pdf]

Supporting Information

Polarization–Tunable Photoelectrochemistry at Individual Anisotropic ReS_2 and Its Homostructure Interfaces

Pelumi Adanigbo[1], Aidan C. Malloy[2], Maha Laiq[1], Lucas Wang[1], Alec G. Freschlin[2], Patrick M. Vora[2,3], Yun Yu*[1,3]

[1] Department of Chemistry and Biochemistry, George Mason University, Fairfax, Virginia 22030, USA

[2] Department of Physics and Astronomy, George Mason University, Fairfax, Virginia 22030, USA

[3] Quantum Science and Engineering Center, George Mason University, Fairfax, Virginia 22030, USA

*Corresponding Author

E-mail: yyu26@gmu.edu

1. **Experimental Methods**

1.1 Chemicals and Materials

Intrinsic ReS_2 crystals and Kish graphite crystals were purchased from 2D Semiconductors. Poly(bisphenol-A-carbonate), potassium iodide, and iodine were purchased from Sigma-Aldrich and used as received. All aqueous electrolyte solutions were prepared with deionized water (Millipore, 18.2 MΩ cm resistivity).

1.2 Sample Preparation and Characterization

ReS_2 and graphene flakes were mechanically exfoliated from the bulk crystals onto SiO_2 (285 nm)/Si wafers (NOVA electronics materials) or indium tin oxide (ITO) coated glass coverslips (SPI supplies) using the "scotch tape" method or gel-film (Gel-pak) assisted method, as previously described.[1] Individual flakes were located by a trinocular compound optical microscopes equipped with CCD cameras. The thickness and the topography of these flakes were determined with atomic force microscopy (NX12, Park System) operated in non-contact mode.

Twisted ReS_2 homostructures were fabricated by vdW assembly using the dry transfer technique[2]. A thin, transparent poly(bisphenol A carbonate) (PC) film was first used to pick up a few-layer graphene flake serving as the bottom contact, followed by the pick-up of a ReS_2 flake as the bottom layer. This ReS_2 flake was then aligned with another ReS_2 flake at the desired twist angle and stacked to form the homostructure. Subsequently, a thick graphite flake (2–10 nm) was picked-up to partially overlap with the graphene contact, serving as an extension. The PC film was then delaminated from the stamp and placed onto a transparent substrate. Finally, Sn/In contacts were established on the graphite extension using a micro-soldering method.[3]

1.3 SECCM Measurement

The SECCM experiments were conducted using a NX12 SICM module (Park Systems) equipped with a scanning probe configuration mounted on an inverted microscope. The incident light illuminated from a fiber-coupled Xenon light source (SLS205, Thorlabs) was directed to the substrate through a 1.45 NA/60× objective lens (Nikon). Band-pass filters (Thorlabs) and neutral-density filters (Thorlabs) were incorporated into the optical path to modulate the excitation wavelength and power, respectively. A glass linear polarizer (Edmund Optics) was mounted on a motorized rotation stage (Thorlabs) to precisely regulate the light polarization.

Single channel nanopipettes were pulled from quartz capillaries using a laser puller (P-2000, Sutter Instrument). The sub-micron tip size was confirmed using an optical microscope and TEM imaging (Figures S12). The nanopipette was filled with electrolyte solution containing KI/I_2. A silver wire coated with AgI was inserted into the channel of the nanopipette, which serves the quasi-reference counter electrodes (QRCE). The nanopipette was positioned over the area of interest with the help of optical microscope. The probe slowly approaches the sample at a speed of 100 nm/s until a >3 pA current was detected due to the contact formed between the meniscus and the sample surface. Local cyclic voltammograms or current–time curves were subsequently recorded at various locations on the sample surface. Data acquisition and polarizer rotation are synchronized by a script that triggers both processes simultaneously.

1.4 Photoconductivity Measurements

Photoconductance measurements were conducted using an electrochemical workstation (CH Instrument). The sample was placed on the inverted microscope, using the same illumination setup as described earlier. Tungsten probes mounted on micropositioners (Everbeing Int'l Corp.) were made in contact with the graphite flakes depicted in **Figure 2**. Electrical bias was applied across arbitrary graphite contacts, and the conductance current was subsequently recorded while the linear polarizer was rotated.

2. Supplementary figures

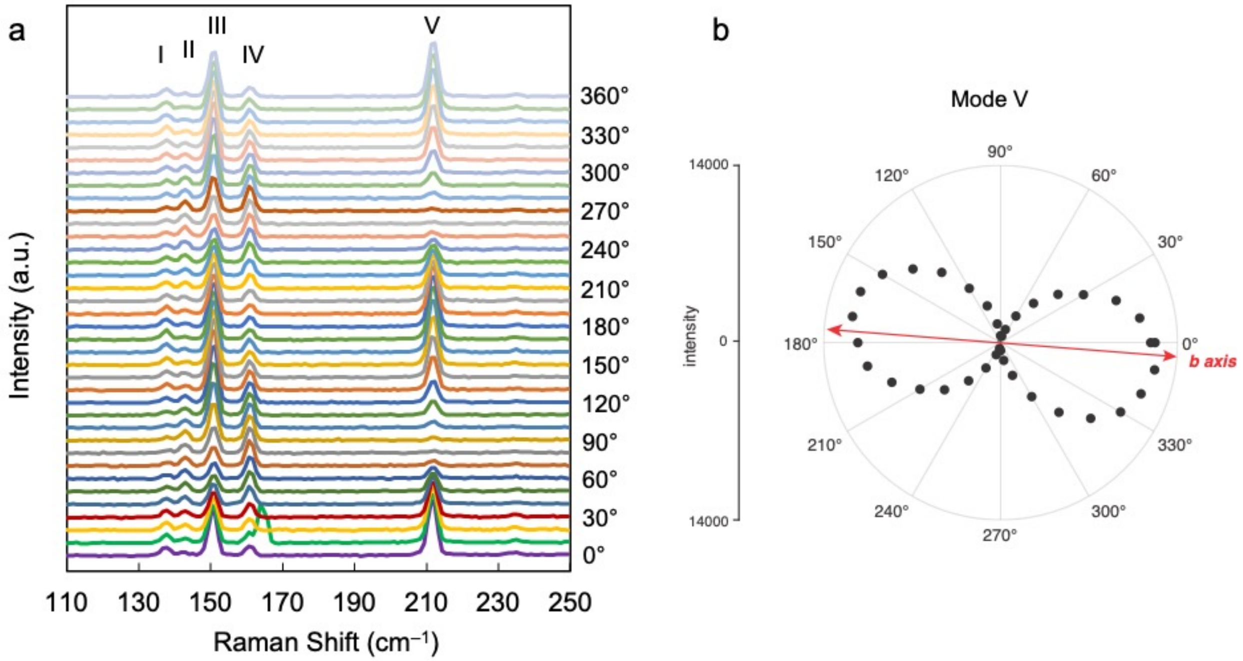

Figure S1. (a) Raman spectra of the ReS_2 sample in Figure 1c with different polarization angles under 532 nm laser excitation. (b) Polar plot of the angle-dependent mode V intensity. The red axis represents the *b*-axis determined from the longer straight edge in the optical image.

Angle-resolved Raman spectroscopy measurements are carried out on ReS_2 flakes exfoliated on an ITO substrate. A linearly polarized 532 nm diode pumped solid state laser is used to excite ReS_2 at atmospheric pressure and temperature. The laser polarization is controlled by a motorized half-wave plate located directly before a 40× magnification microscope objective with a 0.6 NA. Raman scattered light is collected by the same optics and then directed to a series of three Bragg filters which reject the laser line and allow the acquisition of data down to ~10 cm^{-1}. Finally, the Raman scatter is directed to a 500 mm focal length spectrometer with a liquid N_2 cooled charge coupled device.

The detected Raman spectra are fit to a sum of five Lorentzian functions that allow for the determination of individual mode frequencies, amplitudes, and widths as a function of angle. ReS_2 has eighteen Raman-active vibrational modes, all of which are A_g symmetry, giving rise to the present anisotropic behavior[4–7]. We focus on five of these modes that appear in the range of 135 cm^{-1} to 211 cm^{-1}, which we designate as I-V, in accordance with the notation provided in Reference 4. Modes I and II (~135, ~141 cm^{-1}) correspond to vertical distortions of Re atoms. Mode III (~150

cm^{-1}) arises from in-plane motion of Re atoms, acting analogous to E_g modes in other two-dimensional isotropic materials. Modes IV and V (~160, ~211 cm^{-1}) are due to Re in-plane vibrations and E_g-like coupled with S atom displacements. The intensity of mode V at 211 cm^{-1} is highly sensitive to orientation of the anisotropic ReS_2 crystal, being strongest when the laser polarization is aligned with the *b*-axis[4]. To illustrate this, we create a polar plot of the 211 cm^{-1} peak area versus laser polarization angle in Figure S1 along with a red line indicating the orientation of the ReS_2 *b*-axis.

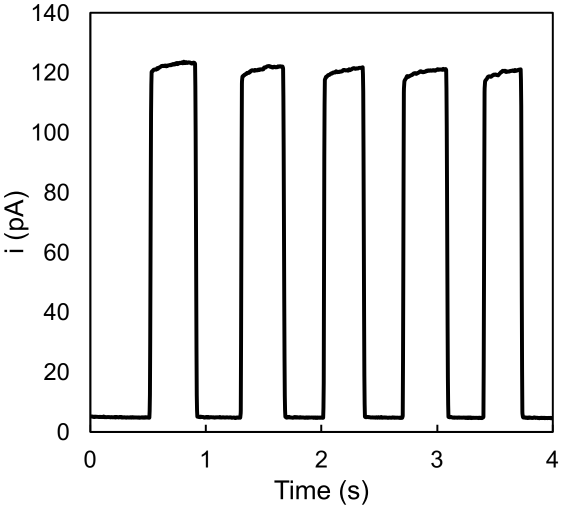

Figure S2. Current vs time trace obtained with an SECCM nanopipette probe as 650 nm excitation is turned on and off. The nanopipette is filled with 0.1 M KI and 10 mM I_2. An electrical bias of 0.4 V vs Ag/AgI is applied to the ReS_2 flake.

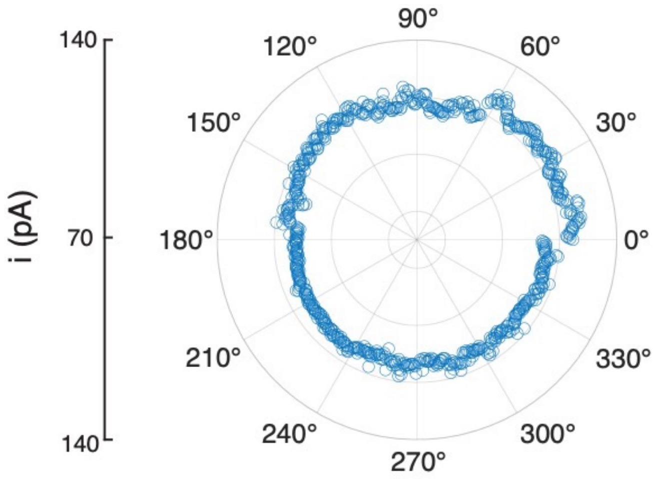

Figure S3. Polar plot of the photoelectrochemical current at a 10-nm-thick 2H-MoS_2 in 0.1 M KI and 10 mM I_2 solution under 290 mW/cm^2 650 nm excitation. An electrical bias of 0.4 V vs Ag/AgI is applied. The circular shape of the response suggests that the photoelectrochemical reaction rate is independent of polarization.

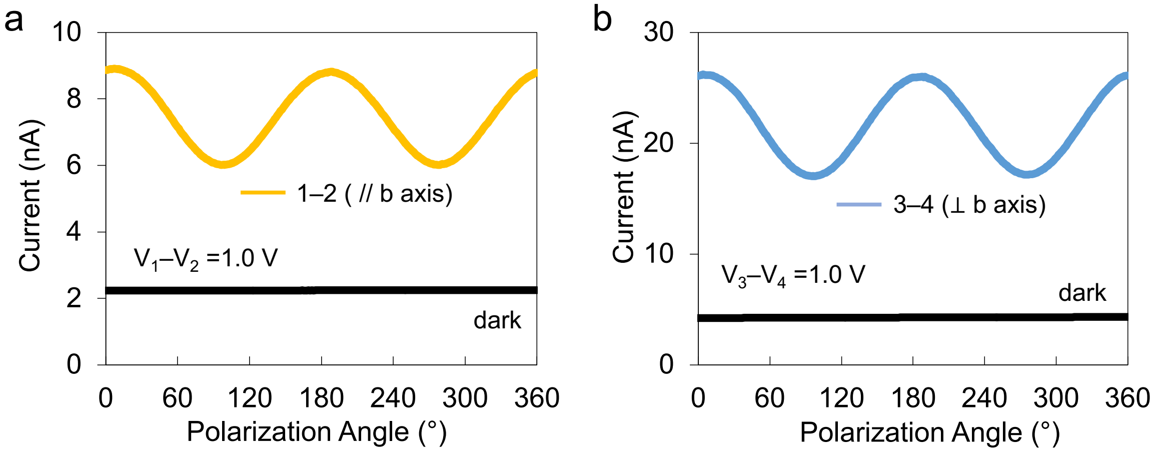

Figure S4. (a) Conductance current collected between probe 1 and probe 2 (Figure 2a) under a voltage of 1.0 V as a function of polarization angle. (b) Conductance current collected between probe 3 and probe 4 under a voltage of 1.0 V as a function of polarization angle. The black curves in both panels indicate a constant dark current background independent of angle.

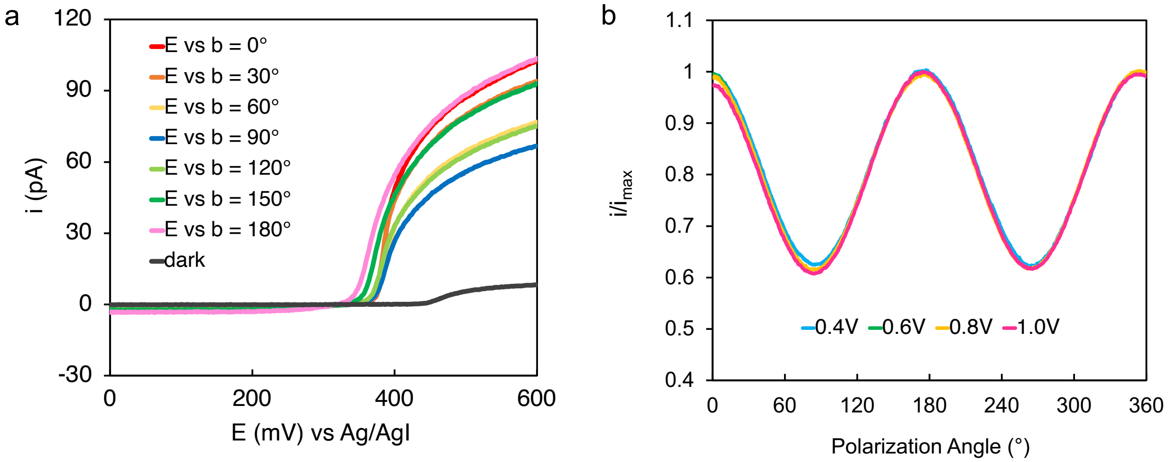

Figure S5. (a) Steady-state voltammograms obtained with an SECCM nanopipette remaining static on an 18-nm-thick ReS_2 surface while the polarizer is rotated from 0° to 180°. (b) Normalized angular-dependent photocurrent response under various constant electric potentials (vs Ag/AgI). The SECCM nanopipette was filled with 0.1 M KI and 10 mM I_2. Scan rate v = 100 mV/s. The ReS_2 was bottom illuminated with 290 mW/cm^2 650 nm light.

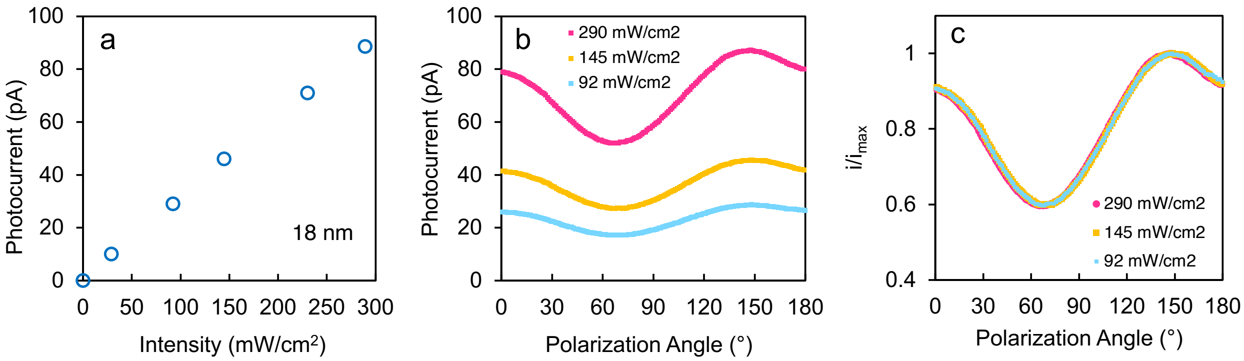

Figure S6. (a) Photocurrent vs intensity plot of an 18-nm-thick ReS_2 flake. (b) Angular dependent photocurrent responses under different light intensities. (c) Normalized angular dependent photocurrents in panel b. The SECCM nanopipette was filled with 0.1 M KI and 10 mM I_2. An electrical bias of 0.4 V vs Ag/AgI was applied. ReS_2 was excited with 650 nm light.

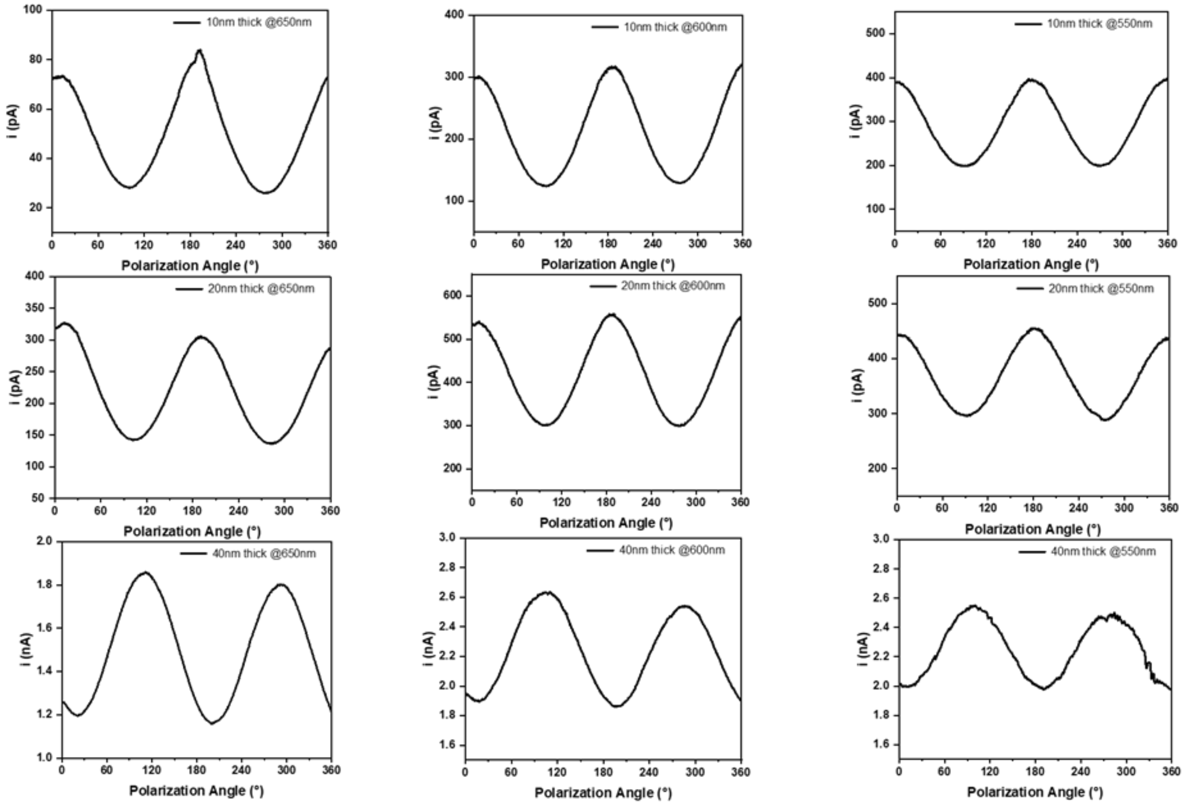

Figure S7. Angular photoelectrochemical current of 0.1M iodide oxidation at a 10-nm, 20-nm, and 40-nm ReS_2 flake under 650 nm, 600 nm, and 550 nm excitation.

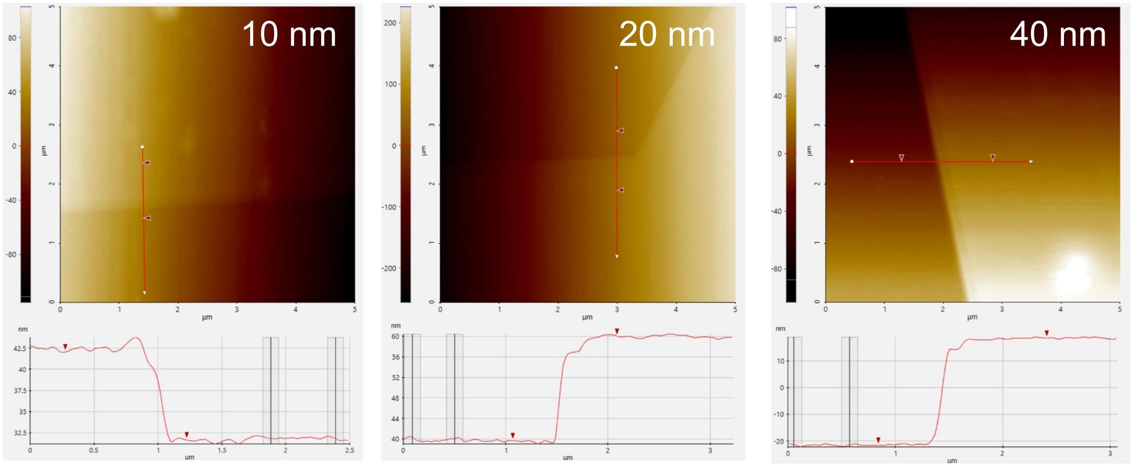

Figure S8. Non-contact topographic AFM images of the 10-nm, 20-nm and 40-nm ReS_2 flake.

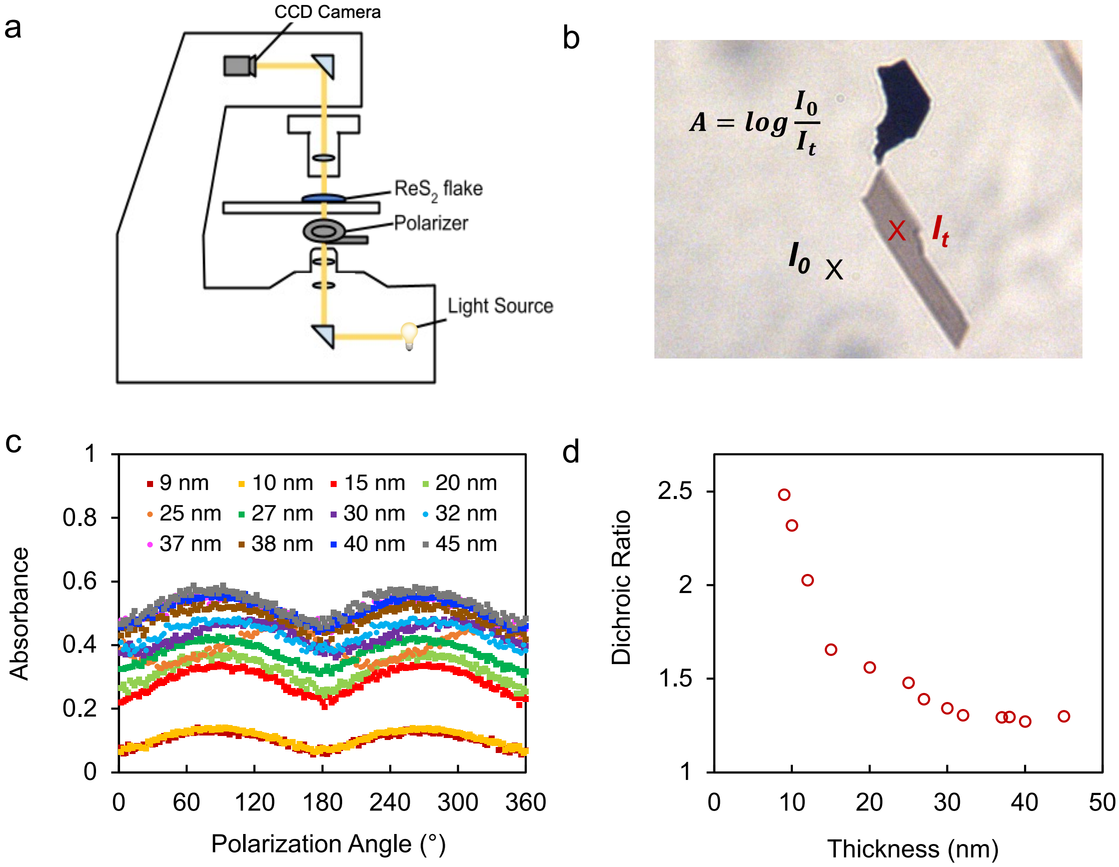

Figure S9: (a) Schematic representation of the experimental setup for absorption measurement. (b) Schematic showing the determination of absorbance, A. (c) Angular dependent absorbance as a function of the flake thickness. (d) Dichroic ratio vs thickness extracted from panel c.

Panel (a) shows that the photoabsorbance of ReS_2 flakes is measured on an inverted microscope by recording how the transmitted light intensity changes with the polarization angle of the incident white light. A linear polarizer is placed in the bottom illumination path. The ReS_2 flake, supported on a transparent substrate, selectively absorbs light depending on how the polarization aligns with its *b*-axis. By rotating the polarizer while keeping the sample and optics fixed, we measure the transmitted intensity through the flake (I_t) at different polarization angles. The absorbance, A, is calculated from the ratio of I_t to the intensity through the bare substrate (I_0) using $A = \log(I_0/I_t)$. This is illustrated in panel (b). Panel (c) and (d) display the absorbance vs angle data of randomly oriented ReS_2 flakes with varying thicknesses from 9 nm to 45 nm. The dichroic ratio, R, is determined by $R = A_{max}/A_{min}$.

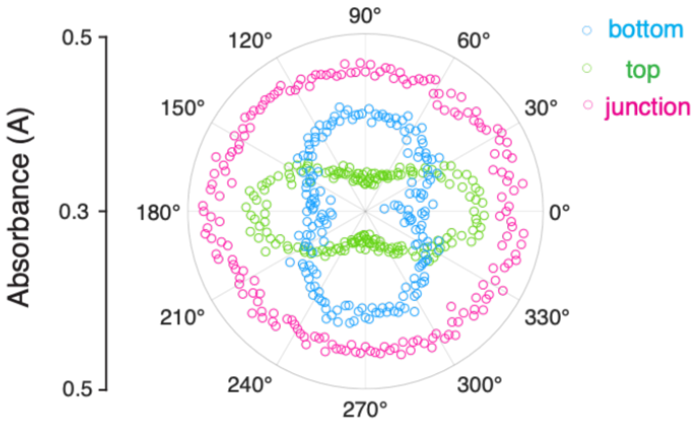

Figure S10. Polar plot of the optical absorbance at different regions within the ReS_2 homostructure, comprised of vertically aligned 33 nm (top) and 37 nm (bottom) flakes. Its photoelectrochemical data is shown in Figure 4d.

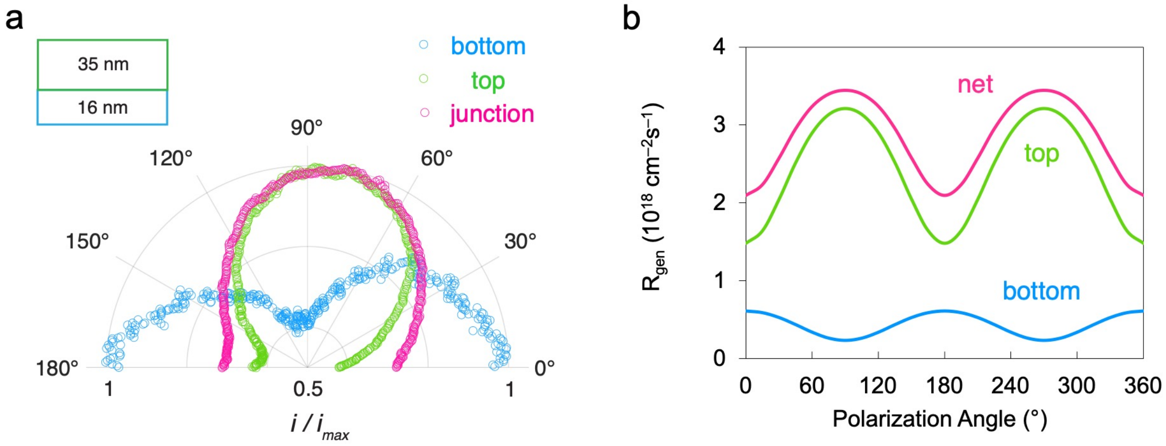

Figure S11. (a) Polar plot depicting the normalized photoelectrochemical current of iodide oxidation at the ReS_2 homostructure, where a 35 nm top layer is vertically stacked on a 16 nm layer. (b) Simulated photocarrier generation rates at the ReS_2 homojunction along with the respective contribution of the top and bottom layer under a photon flux of 10^{19} $cm^{-2}s^{-1}$.

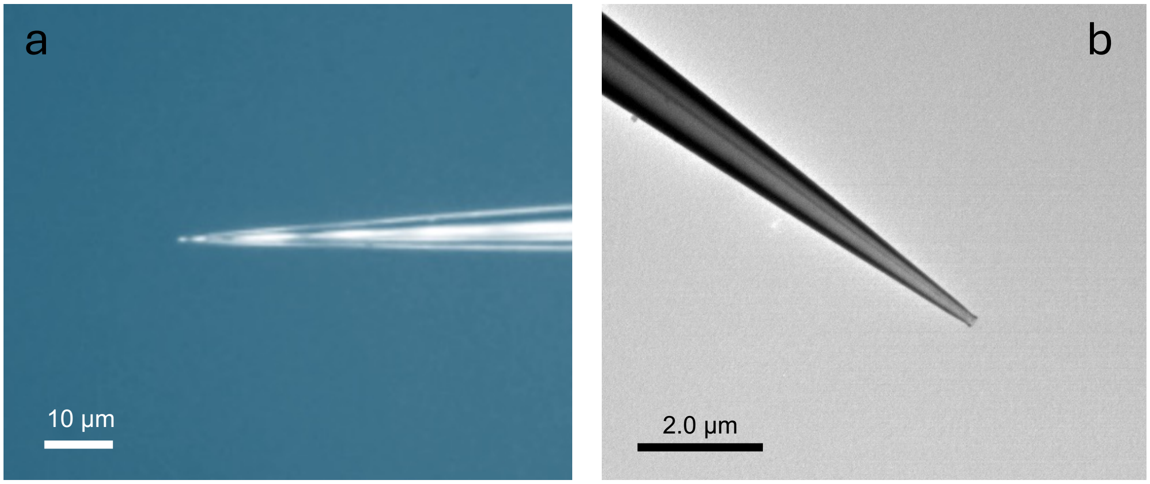

Figure S12. (a) Optical micrograph of a sub-micron nanopipette. (b) TEM image of the same nanopipette tip showing a 160-nm-diameter orifice.

3. Calculation of the generation rate at a vertically stacked ReS_2 junction

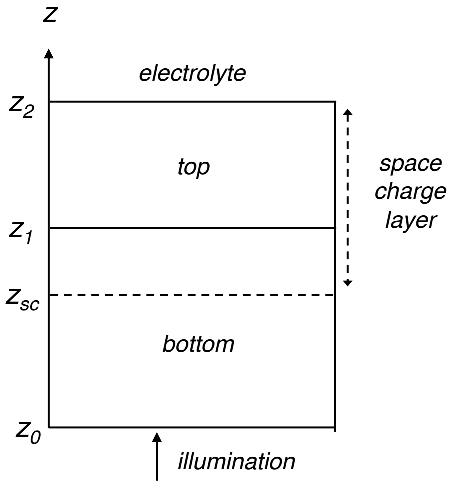

The carrier generation rate at an arbitrary plane of the ReS_2, G (α, z), is given by:

$$G(\alpha, z) = I_0 \alpha e^{-\alpha z} \quad \text{S1}$$

where I_0 is the intensity of the incident light, α is the absorption coefficient.

α_{top} and α_{bottom} are the absorption coefficient of the top layer and bottom layer respectively:

$$\alpha_{top} = \alpha_0 \left[a + bcos^2(\theta - \frac{\pi}{2})\right] \quad \text{S2}$$

$$\alpha_{bottom} = \alpha_0 [a + bcos^2\theta] \quad \text{S3}$$

a and b are constants that describe the in-plane anisotropy of the photo absorption. The values of $a = 0.78 \pm 0.10$, $b = 0.31 \pm 0.18$, and $\alpha_0 = (3.4 \pm 0.7) \times 10^5$ cm^{-1} were estimated from the absorbance data in Figure S9c. θ is the angle of polarization in relative to the b-axis of the bottom layer. A difference of $\pi/2$ is used to account for the 90° alignment. Phase shift is not included in this model.

The contribution of the top layer to the total generate rate is

$$G_{top} = \int_{z_1}^{z_2} I_0' \, \alpha_{top} e^{-\alpha_{top} z} dz \tag{S4}$$

I_0' is the attenuated intensity after light transmitting the bottom layer:

$$I_0' = I_0 e^{-\alpha_{bottom}(z_1 - z_0)} \tag{S5}$$

The contribution of the bottom layer to the total generate rate is

$$G_{bottom} = \int_{z_{sc}}^{z_1} I_0 \, \alpha_{bottom} e^{-\alpha_{bottom} z} dz \tag{S6}$$

z_{sc} represents the boundary of the space charge layer. The thickness of the space charge layer can be determined by:

$$W = z_2 - z_{sc} = \sqrt{\frac{2\varepsilon\varepsilon_0 (V - V_{FB})}{q N_D}} \tag{S7}$$

where ε is the dielectric constant of ReS_2, ε_0 is the vacuum permittivity, V is the applied potential, V_{FB} is the flat band potential ($V_{FB} \approx 0.32$ V vs Ag/AgI based on Figure S5a), q is the elementary charge, and N_D ($N_D \approx 10^{17}$ cm^{-3}) is the electron concentration.

4. Reference

(1) Budania, P.; Baine, P. T.; Montgomery, J. H.; McNeill, D. W.; Neil Mitchell, S. J.; Modreanu, M.; Hurley, P. K. *Micro & Nano Letters* **2017,** *12*, 970-973

(2) Yu, Y.; Zhang, K.; Parks, H.; Babar, M.; Carr, S.; Craig, I. M.; Van Winkle, M.; Lyssenko, A.; Taniguchi, T.; Watanabe, K.; Viswanathan, V.; Bediako, D. K. *Nat. Chem.* **2022,** *14*, 267-273

(3) Girit, Ç. Ö.; Zettl, A. *Appl. Phys. Lett.* **2007,** *91*, 193512

(4) Chenet, D. A.; Aslan, B; Huang, P. Y.; Fan, C.; van der Zande, A.M.; Heinz, T. F.; Hone, J.C. *Nano Lett.* **2015,** *15*, 5667-5672

(5) McCreary A.; Simpson, J. R.; Wang, Y.; Rhodes, D.; Fujisawa, K.; Balicas, L.; Dubey, M.; Crespi, V. H.; Terrones, M.; Hight Walker, A. R. *Nano Lett.* **2017**, 17, 5897–5907

(6) Pradhan, N. R.; McCreary, A.; Rhodes, D.; Lu, Z.; Feng, S.; Manousakis, E.; Smirnov, D.; Namburu, R.; Dubey, M.; Hight Walker, A. R.; Terrones, H.; Terrones, M.; Dobrosavljevic, V.; Balicas, L. *Nano Lett.* **2015**, 15, 8377−8384

(7) Feng, Y. Q.; Zhou, W.; Wang, Y. J.; Zhou, J.; Liu, E. F.; Fu, Y. J.; Ni, Z. H.; Wu, X. L.; Yuan, H. T.; Miao, F.; Wang, B. G.; Wan, X. G.; Xing, D. Y. *Phys. Rev. B: Condens. Matter Mater. Phys*. **2015**, 92, 054110
